# Studying electronic properties in GaN without electrical contacts using *γ*-*γ* vs *e*^−^-*γ* Perturbed Angular Correlations

**DOI:** 10.1038/s41598-019-52098-5

**Published:** 2019-10-31

**Authors:** M. B. Barbosa, J. G. Correia, K. Lorenz, R. Vianden, J. P. Araújo

**Affiliations:** 1 IFIMUP and IN – Institute of Nanoscience and Nanotechnology, Departamento de Física e Astronomia da Faculdade de Ciências da Universidade do Porto, Rua do Campo Alegre 687, 4169-007 Porto, Portugal; 2 C2TN, DECN, Instituto Superior Técnico, Universidade de Lisboa, Lisboa, Portugal; 3 EP Division, CERN, 121 Geneve-23, Switzerland; 4 INESC-MN, IPFN, Instituto Superior Técnico, Universidade de Lisboa, Lisboa, Portugal; 5 Helmholtz-Institut für Strahlen- und Kernphysik, University of Bonn, Bonn, Germany

**Keywords:** Characterization and analytical techniques, Semiconductors

## Abstract

The potential use of combined *e*^−^-*γ* vs *γ*-*γ* Perturbed Angular Correlations (PAC) experiments as a possible alternative to study electronic properties of materials and/or samples where Hall effect measurements are difficult to perform due to low-quality ohmic contacts is here demonstrated using Si- and Zn-doped GaN samples as a showcase example. To do so, the lattice site of implanted ^181^Hf/^181^Ta and the recombination of Ta ionized and excited electronic states were studied as a function of temperature and sample doping in GaN. By combining the *γ*-*γ* and *e*^−^-*γ* PAC results with Density Functional Theory simulations, it was possible to assign a single stable site with a double-donor character for Ta in GaN. A metastable charge state was also identified at particular temperatures using *e*^−^-*γ* PAC. A thermally activated process was observed for the electronic recombination at high temperatures with activation energies of 15(2) meV and 12(1) meV for the Si- and Zn-doped samples, respectively, and attributed to Si shallow donors present in both samples. A reduced number of available electrons was observed in the Zn-doped sample due to donor compensation by the Zn acceptors. At low temperatures, it is suggested that the recombination process occurs via Variable Range Hopping. The doping characteristics of both samples were successfully distinguished.

## Introduction

**Figure 1:**
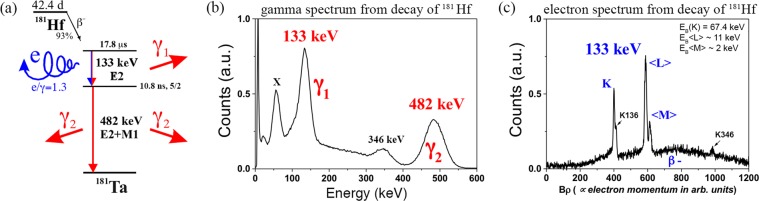
(**a**) Cascade decay of ^181^Hf/^181^Ta and corresponding (**b**) gamma and (**c**) electron spectra, where the first decay of the double cascade occurs by the emission of either a photon or a conversion electron.

**Figure 2:**
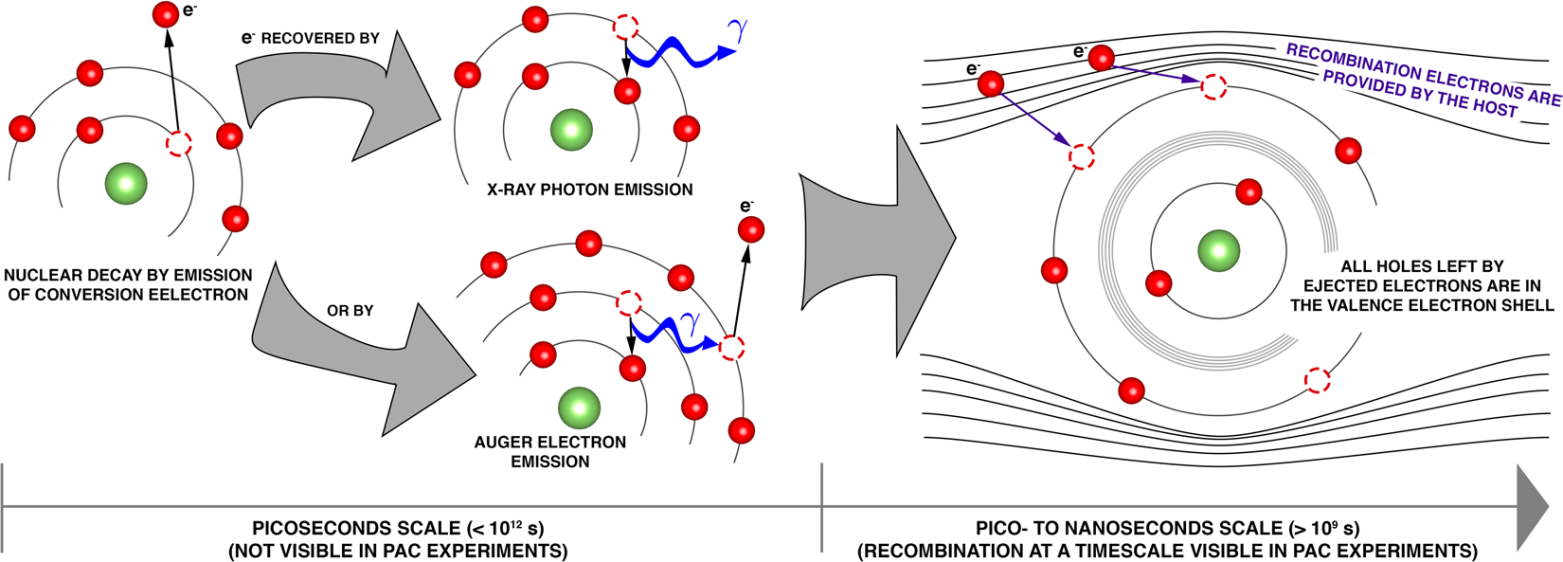
Schematic of the local electronic recombination processes after ejection of a conversion electron due to nuclear decay. The internal electron rearrangement between orbitals, with consequent emission of X-ray and Auger electrons, and their recombination are statistical processes occurring at a time scale below 1 ps, much below the PAC experimental resolution of ~1 ns. Holes left in the valence electron shell after the conversion electron decay are recovered by electrons provided by the host. The number of holes left in the valence electron shells after these core shell recombinations is also statistically distributed for the different probe atoms. If these electron recombination processes are slow enough (nanoseconds scale), they can be observed in the ${e}^{-}$-$\gamma$ PAC experiments.

Hyperfine interactions techniques, such as *γ*-*γ* Perturbed Angular Correlations (PAC), probe energy splittings below 10^−7^ eV, much lower than atomic binding energies, being particularly sensitive to the local electronic density and polarization at a nuclear probe’s site. By measuring the electric quadrupole and magnetic interaction of the nuclear moments with the charge distribution in the surroundings of the probe nuclei, it is possible to find and further characterize point-like defects and the probe’s electronic structure in the host matrix. Complementarily, with the *e*^−^-*γ* PAC technique the measurement starts after the ejection of a conversion electron from the deeper atomic K- or L- shells of the probe element and subsequent X-ray or Auger electron emissions during recombination (see Figs 1 and 2). Consequently, the PAC measurement starts in a perturbed and out-of-equilibrium (maybe) ionized environment. The core recombination process is considered completed when remaining holes are found only in the valence electron shell (Fig. 2, right side). If and how these fluctuating charge distributions are seen in the PAC measurement depends on how fast will be their electronic recombination, which is ruled by both the impurity’s equilibrium charge state (doping character) and the host’s charge carrier density and transport mechanisms. Since the observable of the PAC technique is time differential, the combined study using both *γ*-*γ* and *e*^−^-*γ* techniques allows the local observation of the probe electronic recombination, which is a function, particularly, of charge diffusion and of the stability of localized excited electronic states during the recombination process. Although the combination of both techniques was suggested over 50 years ago by U. Baäverstam, A. Johansson and T.R. Gerholm, fathers of magnetic electron spectroscopy^1^, this idea was not put into practice by the lack of isotopes of appropriate elements with enough purity and quantity (ppm), by poor experimental time resolution (<ns), and still not accessible modeling theory and computing power allowing to unambiguously analyze and interpret the experimental data. From the experimental point of view, the ISOLDE-CERN laboratory provides now short-lived radioactive probes with enough purity and quantity, and there are the most efficient *γ*-*γ* and *e*^−^-*γ* PAC spectrometers ever built, including the only functional P. Kleinheinz and K. Siegbahn spectrometer with two magnetic lenses and two BaF_2_ detectors^2,3^. From the data analysis point of view, the in-house development of the program *PACme* (to be published) was the turning point to analyze *γ*-*γ* and *e*^−^-*γ* combined experiments in a truly informative manner.

One suitable isotope allowing to combine *γ*-*γ* with *e*^−^-*γ* PAC is ^181^Hf (*t*_1/2_ = 42.4 days) decaying by *β*^−^ to a first 615 keV long-lived metastable state (*t*_1/2_ = 17.6 μs) at ^181^Ta. Stable ^181^Ta is achieved following a double cascade whose intermediate level has spin 5/2 and *t*_1/2_ = 10.8 ns (Fig. 1). The first decay of this cascade may occur by emission of a photon or by emission of a conversion electron with a total internal-conversion coefficient of $\alpha =\tfrac{e}{\gamma }=\tfrac{{\rm{number}}\,{\rm{of}}\,\text{de}-\text{excitations}\,{\rm{via}}\,{\rm{electron}}\,{\rm{emission}}}{{\rm{number}}\,{\rm{of}}\,\text{de}-\text{excitations}\,{\rm{via}}\,{\rm{gamma}}\,{\rm{emission}}}=1.265$^4^. This makes ^181^Hf/^181^Ta a good probe to combine *γ*-*γ* PAC using *γ*_1_ (133 keV) and *γ*_2_ (482 keV) with *e*^−^-*γ* PAC using the conversion electron $\langle L\rangle$ with average energy ~122 keV (133 keV–11 keV) and *γ*_2_ (see Fig. 1 for the gamma and electron spectra).

*γ*-*γ* and *e*^−^-*γ* PAC experiments were then performed in Si- and Zn-doped GaN samples as a function of temperature after implantation of ^181^Hf/^181^Ta to study the probe’s atomic location and the charge carrier transport in each sample. Density Functional Theory simulations were used to better interpret the experimental observations. GaN is a wide band gap semiconductor (3.503(5) eV^5^) especially useful in the development of blue- and UV-LEDs^6–8^. Si is known to be a very good n-type dopant in GaN by substituting for Ga and introducing a shallow donor level into the band gap^9^. On the other hand, Zn substitutes for Ga and introduces a deep acceptor level above the top of the valence band maximum, being responsible for the blue luminescence peaking at 2.9 eV that is often observed in PL spectroscopy^10–12^. The deep acceptor is commonly used to compensate unintentional donor defects to manufacture semi-insulating GaN^13^. Implantation studies of the double acceptor Ta in GaN have been done before^8,14^, but we provide now a new and detailed microscopic investigation enlightening both the Ta impurity states and their dependence on the properties of the pre-doped GaN host. This work showcases the potential use of combined *γ*-*γ* vs *e*^−^-*γ* PAC experiments as a possible alternative to study the electronic properties of materials without electrical contacts.

## Results

### Perturbed angular correlation experiments

*γ*-*γ* and *e*^−^-*γ* PAC measurements were performed as a function of temperature – between room temperature and 10 K – in two ~11 μm thick GaN samples on sapphire substrates and doped with Si (concentration ${N}_{d}-{N}_{a}=(1\,-\,3)\times {10}^{18}\,{{\rm{cm}}}^{-3}$) and Zn (high resistivity $> \,1\times {10}^{5}\,\Omega \,{\rm{cm}}$), respectively. *e*^−^-*γ* PAC experiments were further performed (under vacuum) from room temperature up to ~500 K.

**Figure 3:**
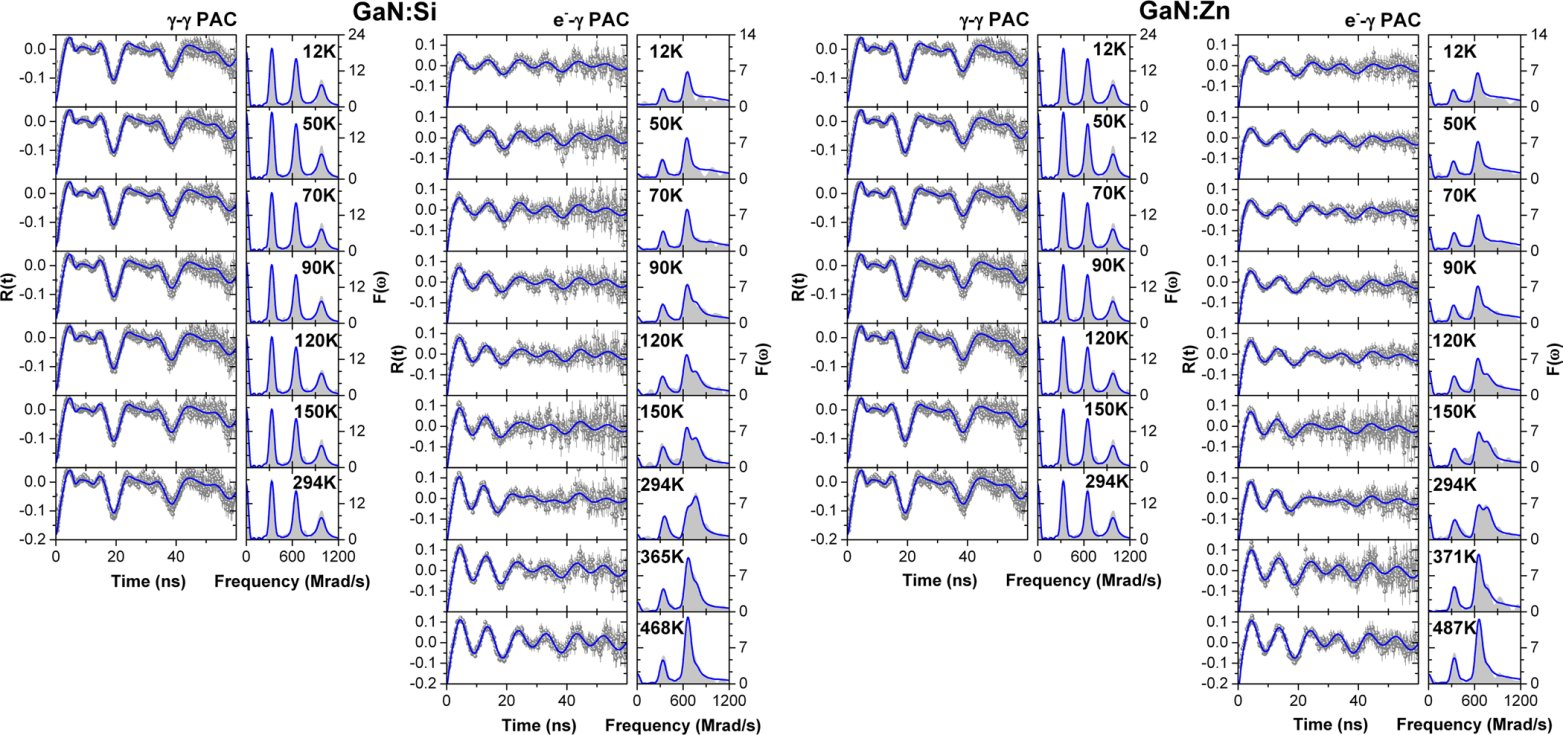
(left) $\gamma$-$\gamma$ and (right) ${e}^{-}$-$\gamma$ PAC spectra (and corresponding Fourier transforms) as a function of temperature after implantation of ^181^Hf/^181^Ta and annealing at 1000 °C for 10 minutes in single crystalline samples of GaN(Si-doped) and GaN(Zn-doped).

Figure 3 shows the resulting PAC *R*(*t*) observable spectra and respective Fourier transforms for each sample, including the *γ*-*γ* and the *e*^−^-*γ* measurements, whilst Tables 1 and 2 summarize the fitting parameters.

**Table 1:** *γ*-*γ* PAC fitting parameters obtained for the measurements as a function of temperature in GaN(Si-doped) and GaN(Zn-doped).

Sample	T (K)	Fraction 1					Fraction 2			
		%	*ω*_0_	*η*	*α*	FWHM	%	*ω*_0_	*η*	FWHM
GaN:Si	12	95.7 (1)	325.1 (4)	0.14 (1)	56.5 (1)°	22.1 (4)	4.3 (1)	978 (2)	0.0	146 (4)
	50		325.1 (4)	0.14 (1)						
	70		324.4 (3)	0.14 (1)						
	90		324.4 (3)	0.14 (1)						
	120		324.7 (5)	0.14 (1)						
	150		324.8 (2)	0.14 (1)						
	294		324.7 (2)	0.14 (1)						
GaN:Zn	12	95.9 (2)	323.2 (6)	0.14 (1)	59.8 (1)°	25.9 (5)	4.1 (2)	971 (13)	0.0	536 (37)
	50		322.2 (5)	0.16 (1)						
	70		322.6 (7)	0.15 (1)						
	90		322.0 (5)	0.16 (1)						
	120		322.0 (7)	0.16 (1)						
	150		322.4 (5)	0.15 (1)						
	294		322.6 (2)	0.14 (1)						

Two different fractions were considered, each corresponding to a different probe’s environment characterized by a single EFG. The first fraction corresponds to probes in a stable site whereas the second fraction corresponds to non-annealed probes and is treated as a polycrystalline environment. The quadrupole frequency ${\omega }_{0}$ and the full width at half maximum for the Lorentzian distribution (FWHM) are expressed in Mrad/s and the asymmetry parameter $\eta$ is dimensionless. *α* is the EFG’s azimuth angle.

**Table 2:** *e*^−^-*γ* PAC fitting parameters obtained for the measurements as a function of temperature in GaN(Si-doped) and GaN(Zn-doped).

Sample	T (K)	State 1			State 2			State 3			Transition Rates		
		*ω*_0_	*η*	FWHM	*ω*_0_	*η*	FWHM	*ω*_0_	*η*	FWHM	1 → 2	1 → 3	2 → 3
GaN:Si	12	423 (19)	0.0 (2)	400 (56)	388 (2)	0.00 (2)	18 (3)	322 (2)	0.12 (7)	22 (4)	0	221 (18)	—
	50							319 (2)	0.10 (5)		0	317 (27)	—
	70							322 (2)	0.16 (5)		0	342 (33)	—
	90							319 (2)	0.0 (2)		194 (33)	331 (29)	38 (3)
	120							315 (2)	0.0 (2)		333 (52)	439 (46)	
	150							316 (2)	0.10 (6)		738 (169)	614 (122)	
	294							317 (1)	0.18 (5)		2074 (356)	386 (99)	
	365							327 (1)	0.00 (5)		1172 (119)	1428 (115)	
	468							328 (1)	0.00 (2)		617 (107)	2293 (358)	
GaN:Zn	12	423 (15)	0.0 (4)	400 (11)	388 (3)	0.00 (3)	18 (5)	313 (2)	0.0 (1)	22 (5)	0	199 (14)	—
	50							315 (1)	0.0 (1)		0	244 (11)	—
	70							319 (1)	0.13 (5)		0	244 (16)	—
	90							314 (2)	0.0 (1)		68 (20)	265 (18)	42 (5)
	120							314 (2)	0.1 (1)		226 (32)	335 (35)	
	150							317 (4)	0.0 (1)		345 (91)	292 (63)	
	294							315 (3)	0.0 (1)		730 (101)	346 (63)	
	371							322 (1)	0.10 (6)		341 (92)	815 (136)	
	487							325 (1)	0.09 (3)		230 (52)	1033 (106)	

Three different states described by a single electric quadrupole interaction each were considered. The transition rates between them are expressed in MHz. The quadrupole frequency ${\omega }_{0}$ and the full width at half maximum for the Lorentzian distribution (FWHM) are expressed in Mrad/s and the asymmetry parameter $\eta$ is dimensionless.

E. Alves *et al*.^8^ showed that Hf is incorporated into substitutional Ga sites in GaN immediately after the implantation. In the *γ*-*γ* PAC measurements of both samples it is indeed possible to see that a single environment is observed (described by a quadrupole frequency ${\omega }_{0}\sim 325\,{\rm{Mrad}}/{\rm{s}}$, and an asymmetry parameter $\eta \sim 0.14$) – a unique electric field gradient (EFG) results in 3 observable frequencies for a spin-5/2 nuclear level – in accordance with the previous reports^8,14^. Nonetheless, a small fraction (~5%) characterized by a very broad EFG (Lorentzian distribution with large FWHM) is also present, representing a small amount of implantation defects that were not properly annealed. More importantly, within the range of measured temperatures, it can be seen that no significant changes occur in the spectra, meaning that the electronic environment of the Ta probe and the GaN lattice stays particularly stable.

On the contrary, a different picture is seen in the *e*^−^-*γ* PAC measurements, where a clear dependence of the *R*(*t*) function as a function of temperature is visible. Since both *γ*-*γ* and *e*^−^-*γ* PAC experiments are performed in the same samples, the temperature dependence of the R(t) function observed in the *e*^−^-*γ* PAC experiments must be triggered by the emission of the internal conversion L-shell electron and the subsequent Ta electronic shells recombination process. After the emission of the conversion electron, during the recombination of the atomic shells X-rays or Auger electrons might be emitted, creating a distribution of ionized states at the Ta probe. Consequently, while electronic recovery is partially achieved / completed the charge distribution around a probe nuclei is probabilistically different from probe atom to probe atom, depending on the initial distribution of charge states and on the possibility to rapidly find recovery electrons from their neighborhood. Therefore, the initial observable state describing all probes (State 1) is characterized by a distribution of EFGs which can be approximately described by a single central EFG (*ω*_0_ = 423 Mrad/s, *η* = 0.0) with a very broad distribution (large FWHM) to account for the differences in the initial EFGs of the different probe nuclei. Then, a transition occurs to the final stable state (State 3, *ω*_0_ = 328 Mrad/s, *η* = 0.0), without possibility of return to the initial state (unidirectional transition).

To properly analyze this data, the fitting program *PACme* (to be published) that incorporates the theory of stochastic processes applied to dynamic transitions in PAC, as described by Winkler and Gerdau^15^, was used.

Table 2 shows the fitting parameters obtained after analyzing the spectra. Interestingly, between 120 K and 294 K a metastable Ta state is observed (State 2) (*ω*_0_ = 388 Mrad/s, *η* = 0.0) in addition to the final stable state. In fact, around 294 K this metastable state is more visible than the final stable state for the GaN:Si sample. It was then possible to determine the EFG corresponding to each existing Ta electronic state and the transition rates between them at each measuring temperature. We particularly emphasize the fact that each specific EFG, characteristic for a specific charge distribution, barely changes with temperature while the transition rates from one state to another strongly depend on temperature. Also, only the relevant transitions ${\rm{State}}\,1\to {\rm{State}}\,2$, ${\rm{State}}\,1\to {\rm{State}}\,3$ and ${\rm{State}}\,2\to {\rm{State}}\,3$ are listed in Table 2, where the remaining possible transitions are null throughout the entire experiments.

### Density functional theory simulations

**Figure 4:**
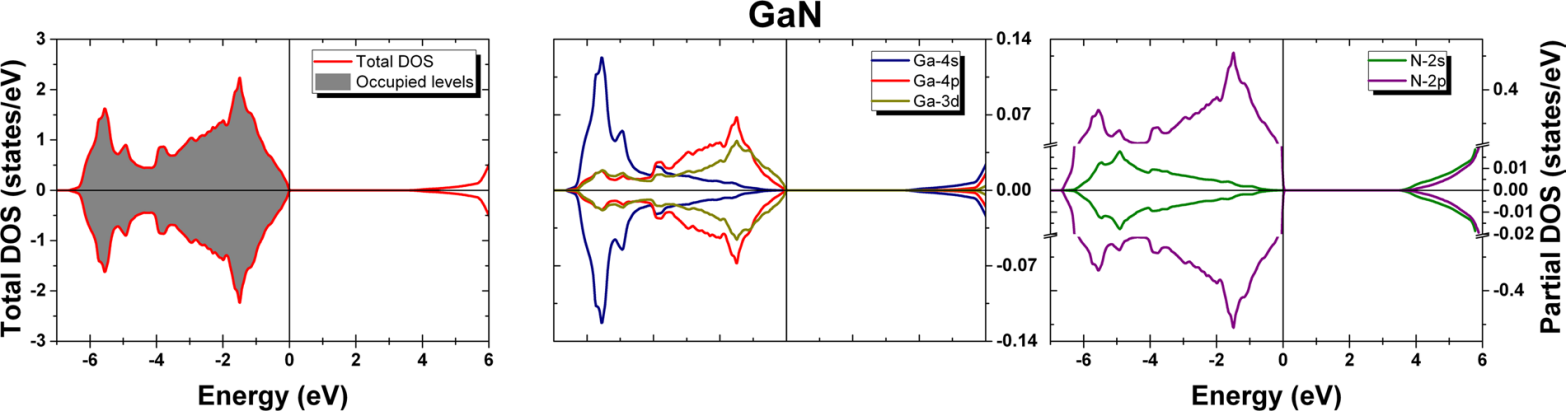
Total and partial density of states (DOS) from GaN. The top valence band is set at 0 eV.

In order to better understand the lattice site and the charge state of the Ta probes in the GaN samples during the PAC measurements, DFT simulations were performed for pure GaN and for GaN supercells with Ta impurities (considering the GaN wurtzite structural parameters as found in the work of Miwa *et al*.^6^, $a=3.146\,{\rm{\AA }}$, $b=3.146\,{\rm{\AA }}$, $c=5.125\,{\rm{\AA }}$). The density of states for pure GaN (Fig. 4) shows the top valence band dominated by N 2*p* states, as well as the chemical bond regions and hybridization of the Ga 4*p* and N 2*p* states between −1.0 and −2.0 eV and of the Ga 4*s* and N 2*p* states between −5.5 and −6.5 eV, in accordance with earlier works^16^. The main contribution from the Ga 3*d* states occurs deeper in the valence band (between −11.3 eV and −13.3 eV, not shown here) but a small contribution is also present at the top valence band.

**Figure 5:**
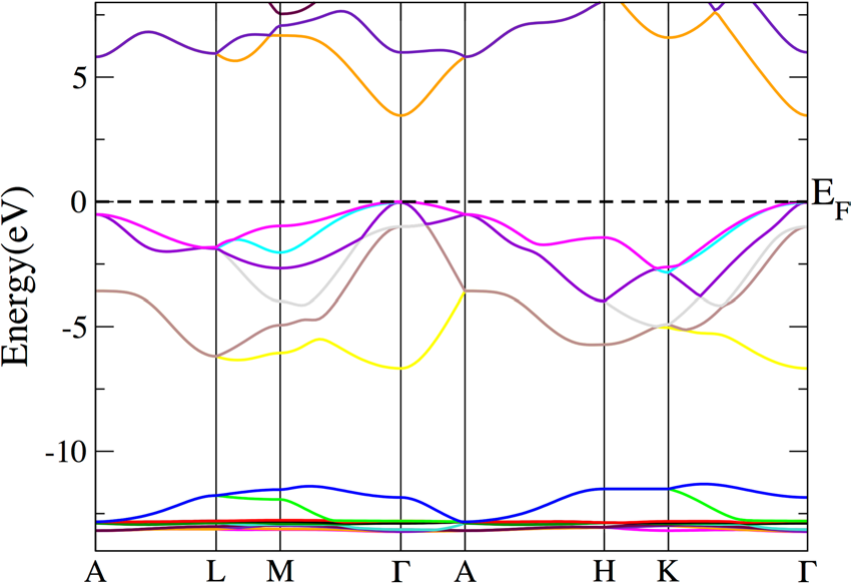
Band structure of GaN in the wurtzite structure. The Fermi energy is set to 0 eV. The k-point labels are named as in ref.^32^ for the hexagonal lattice and following the same path as in ref.^6^.

A band gap of 3.46 eV was obtained, which is in very good agreement with the experimental value of 3.503(5) eV^5^, having an accuracy comparable to simulations using the OEPx(cLDA) + *G*_0_*W*_0_ method (3.24 eV)^17^ and a much better agreement with experiment than previous simulations using the WC-GGA + U functional (2.40 eV)^16^. The bandstructure is characterised by a direct band gap at the $\Gamma$ point, as expected for the wurtzite structure of GaN (Fig. 5). Since the bottom of the conduction band is well approximated by a parabolic dispersion relation (despite a slight anisotropy due to the reduced lattice symmetry^18^), an electron effective mass of 0.24 *m*_e_ was found by parabolic fitting the conduction band minimum (CBM), in good accordance with the experimental electron effective mass of 0.20 *m*_e_^18^.

Since the EFG is very sensitive to the structural parameters (specially in the case of impurity atoms in supercells), a volume optimization was performed to test the reported lattice parameters before calculating the supercells with the Ta probe and the parameters corresponding to the lowest energy are $a=b=3.2187\,{\rm{\AA }}$ and $c=5.2415\,{\rm{\AA }}$. The density of states and band structure obtained for these optimized lattice parameters are similar to the previous results (Figs 4 and 5) but the top valence band is 0.44 eV narrower and the band gap is 0.4 eV smaller. However, the lattice parameters changed considerably, which could lead to substantial changes in the EFG at the Ta site in a supercell. Therefore, both sets of lattice parameters were considered to study the environment around the Ta impurity in GaN.

In order to simulate an isolated Ta impurity, a 3 × 3 × 2 supercell of GaN was constructed. Since Ta and Ga usually have different cation-like oxidation states (+3 for Ga and +5 for Ta), i.e., Ta vs Ga has 2 extra valence electrons, the following different positions and charge states were considered for the Ta in the supercell:

1. Ta at the Ga site with the following charge states:
  - $${{\rm{Ta}}}_{{\rm{Ga}}}^{0}$$: neutral,
  - ${{\rm{Ta}}}_{{\rm{Ga}}}^{2+}$: two electrons removed from the system,
  - ${{\rm{Ta}}}_{{\rm{Ga}}}^{1+}$: one electron removed from the system.
2. Ta at the interstitial position between the tetrahedra formed by a centered Ga atom with N atoms in the vertices, with the following charge states:
  - $${{\rm{Ta}}}_{i}^{0}$$: neutral,
  - ${{\rm{Ta}}}_{i}^{5+}$: five electrons removed from the system,
  - ${{\rm{Ta}}}_{i}^{4+}$: four electrons removed from the system.

Table 3 summarizes the EFGs at the Ta probe calculated for each of the positions and charge states, together with the experimental EFGs observed in the PAC experiments.

**Table 3:** Calculated z-component of the EFG tensor in the principal components axis (in units of V/Å^2^) for each position and charge state of the Ta probe using the reported^6^, optimized and best lattice parameters, as well as the experimental values obtained in the *e*^−^-*γ* PAC measurements (considering the electric quadrupole moment $Q=2.35(6)\,{\rm{b}}$^4,33^).

		*V*_*zz*_ (V/Å^2^)		
		Reported lat.	Optimized lat.	Best lat.
Simulated	${{\rm{Ta}}}_{{\rm{Ga}}}^{0}$	113.3	65.4	114.2
	${\bf{T}}{{\bf{a}}}_{{\bf{G}}{\bf{a}}}^{1{\boldsymbol{+}}}$	63.3	45.4	**73**.**0**
	${\bf{T}}{{\bf{a}}}_{{\bf{G}}{\bf{a}}}^{2{\boldsymbol{+}}}$	47.2	37.1	**62**.**0**
	${{\rm{Ta}}}_{i}^{0}$	134.2	160.1	—
	${{\rm{Ta}}}_{i}^{4+}$	155.6	240.2	—
	${{\rm{Ta}}}_{i}^{5+}$	226.3	257.6	—
Exp.	**State 2**	**72 (2)**		
	**State 3**	**61 (2)**		

The asymmetry parameter is zero for all cases but the experimental State 3 $(\eta \in [0.0,0.18])$.

**Figure 6:**
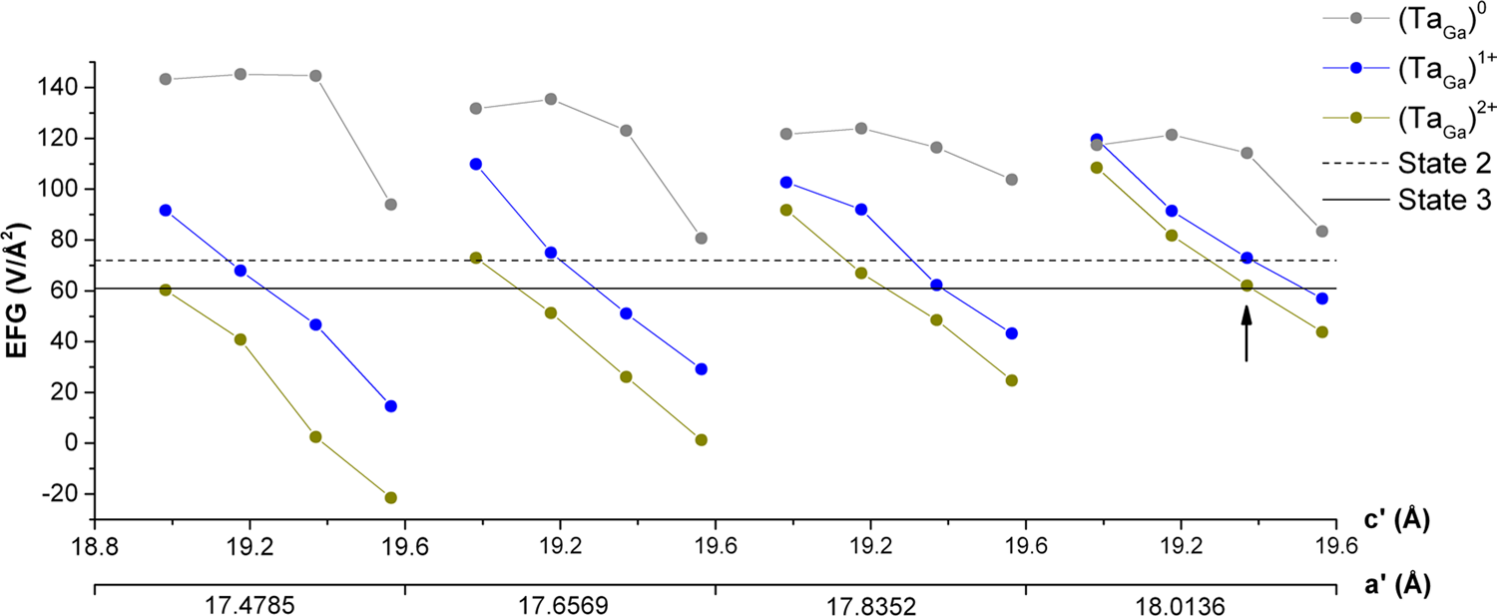
Calculated EFG at the ${{\rm{Ta}}}_{{\rm{Ga}}}^{0}$, ${{\rm{Ta}}}_{{\rm{Ga}}}^{1+}$ and ${{\rm{Ta}}}_{{\rm{Ga}}}^{2+}$ sites as a function of the supercell’s lattice parameters *a*′ and *c*′. EFGs corresponding to State 2 and State 3 are from the *e*^−^-*γ* PAC experiments. The arrow shows the best fit between experimental and calculated EFGs.

It can be seen that only the EFGs corresponding to the Ta atom at the Ga site are comparable to the experimental EFGs obtained from the PAC experiments (the remaining ones are much higher), but there is no exact match. Therefore, other sets of parameters were tested for the supercell considering a variation of −2%, −1%, 0% and 1% around the original lattice parameters. In Fig. 6, the variation of the EFG for each charge state can be seen, where the most pronounced variation is the decrease of the EFGs for increasing *c*. More importantly, it can be seen that the EFG for the neutral state of Ta is always higher than any of the experimental EFGs, whilst the other two charge states have several crossovers with the experimental values. In fact, the EFGs from the charge states of one set of parameters match very well the experimental EFGs (see arrow in Fig. 6). This enables us to conclude that the Ta_Ga_ in the charge state ${{\rm{Ta}}}_{{\rm{Ga}}}^{1+}$ corresponds to the measured EFG assigned as State 2 while the EFG assigned as State 3 corresponds to Ta_Ga_ in the charge state ${{\rm{Ta}}}_{{\rm{Ga}}}^{2+}$.

**Figure 7:**
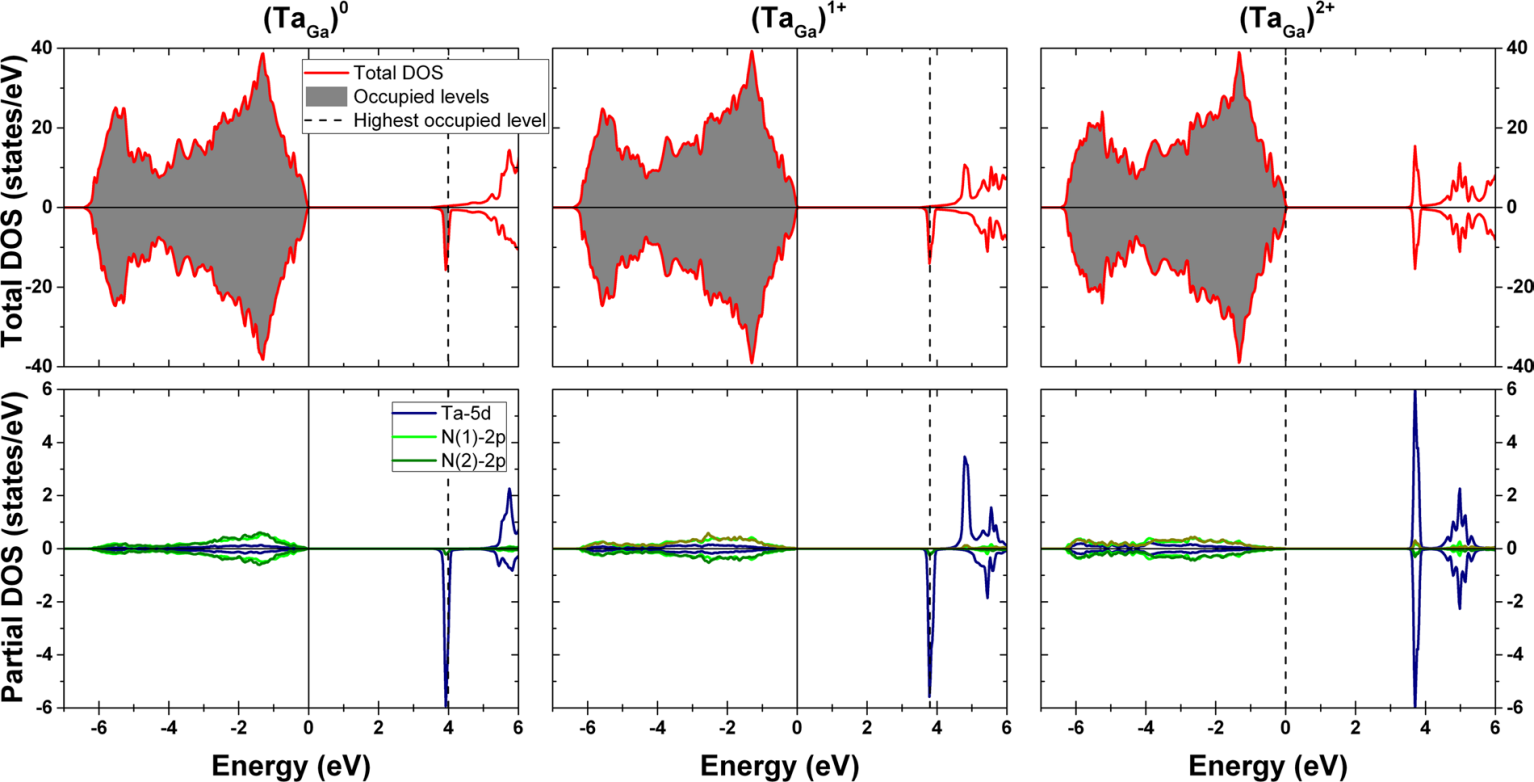
Total and partial density of states (DOS) from the GaN supercells with ${{\rm{Ta}}}_{{\rm{Ga}}}^{0}$, ${{\rm{Ta}}}_{{\rm{Ga}}}^{1+}$ and ${{\rm{Ta}}}_{{\rm{Ga}}}^{2+}$. The top valence band of pure GaN is set at 0 eV (full line) and the energy of the highest occupied level is represented by a dotted line. For ${{\rm{Ta}}}_{{\rm{Ga}}}^{2+}$, both lines are coincident.

Figure 7 shows the density of states (DOS) for all charge states of Ta_Ga_. The presented values use the lattice parameters whose simulated EFGs best match the experimental EFGs, however we should point out that almost indistinguishable DOS variations are observed if using the original lattice parameters. It can be seen in the total DOS that replacing a Ga atom by a Ta^0^ probe leaves the basic electronic structure of GaN unaltered but a partially filled impurity band appears in the bottom of the conduction band. By integrating the unfilled region between the beginning of this peak and the highest occupied state, a value of 1.9 electrons is obtained, revealing a double-donor character for Ta in this material. The atom-resolved partial DOS projected at the Ta and neighboring N atoms (Fig. 7) shows that the impurity band is mainly composed of Ta-5*d* orbitals, with a very small contribution from N-2*p* orbitals. The Ta-4*f* orbitals are too deep in energy (≈−20 eV) and therefore do not play a significant role in this case. A similar behavior is observed for the charge states ${{\rm{Ta}}}_{{\rm{Ga}}}^{1+}$ and ${{\rm{Ta}}}_{{\rm{Ga}}}^{2+}$, but the integration of the impurity band in the former case gives 1.0 electron and it is completely empty in the latter case.

### Model for the EFG temperature dependence in *e*^−^-*γ* PAC

After identifying ${{\rm{Ta}}}_{{\rm{Ga}}}^{1+}$ as the intermediate state (State 2) and ${{\rm{Ta}}}_{{\rm{Ga}}}^{2+}$ as the final state (State 3) in the *e*^−^-*γ* PAC experiments (${{\rm{Ta}}}_{{\rm{Ga}}}^{2+}$ is also the only stable state present in the *γ*-*γ* PAC experiments), the temperature dependence of the transition rates must be understood. Since the essential EFGs phenomenology is due to the electronic recovery of the distribution of ionized initial states to any of the observable stable/metastable states, we can look at this process as equivalent to the “decay” of State 1 and therefore analyze the sum of all transition rates from this state to all other states: ${\Gamma }_{1}={\omega }_{1\to 2}+{\omega }_{1\to 3}$, where ${\omega }_{i\to j}$ is the transition rate from state *i* to state *j*. In fact, the inverse of this sum (${\tau }_{1}=1/{\Gamma }_{1}$) can be interpreted as the characteristic *mean life* time of State 1^15^ (e.g., ${\tau }_{1}\approx 1\,{\rm{ns}}$ at 294 K).

**Figure 8:**
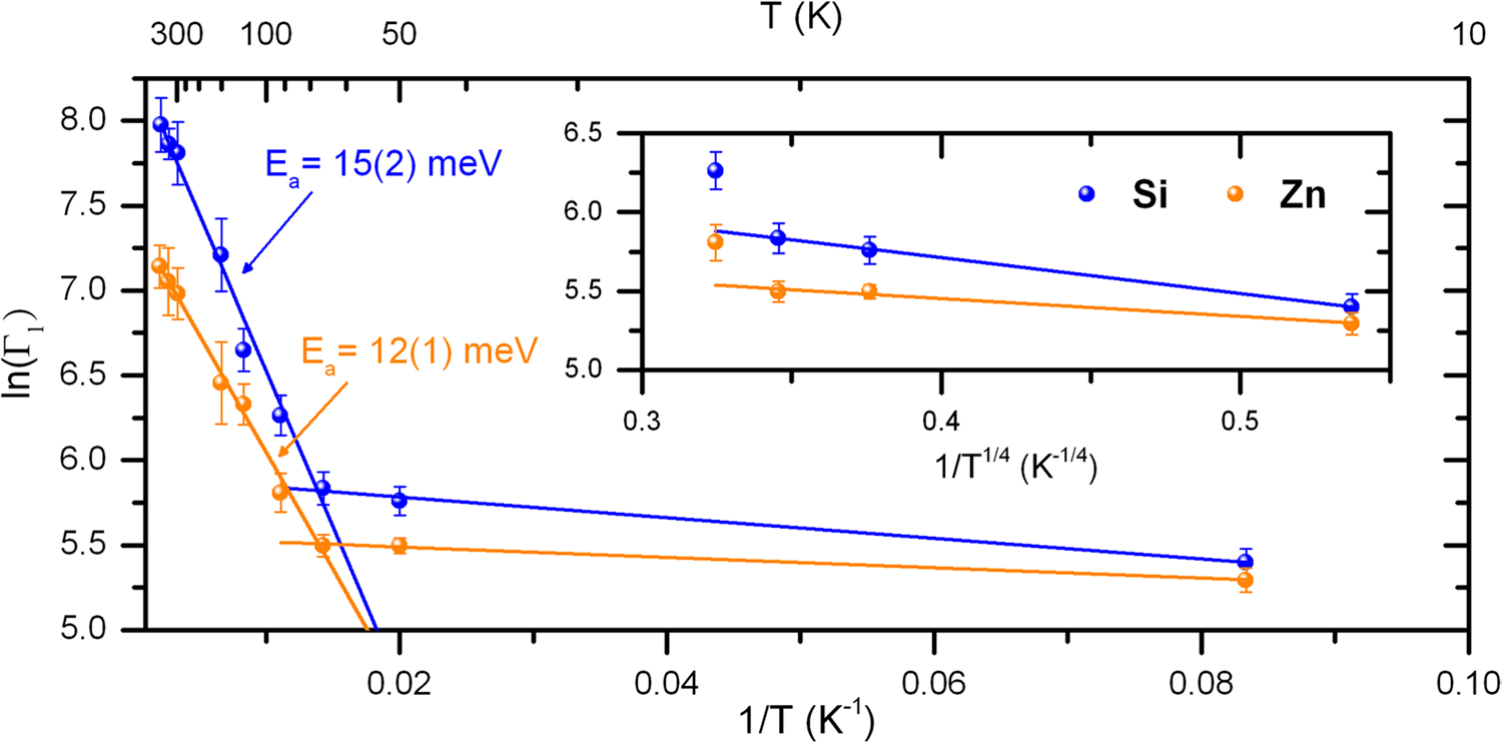
Arrhenius plot of ${\Gamma }_{1}(T)$ in logarithmic scale and the corresponding fit for the two different temperature regimes. The error bars were calculated considering $\Delta \,\mathrm{ln}(x)=\Delta x/x$. In the inset, the transition rates between 12 K and 70 K are plotted as a function of ${T}^{-1/4}$, characteristic for Variable Range Hopping.

In the reasonable attempt to try to fit the data with an Arrhenius temperature dependence for ${\Gamma }_{1}(T)$ (expected for a thermally activated process), it can be seen in Fig. 8 that two different regimes are present in both samples, one for temperatures below 70 K and another for temperatures above. In the higher temperatures regime, a linear behavior is observed in the logarithmic scale plot with associated activation energies of 15(2) meV and 12(1) meV for the GaN:Si and GaN:Zn samples, respectively.

Götz *et al*.^9^ performed variable temperature Hall effect measurements (from 80 K to 500 K) and low-temperature (2 K) photoluminescence spectroscopy (PL) in n-type GaN films which were either unintentionally doped or Si doped. They found that the n-type conductivity for both types of samples was dominated by a donor with an activation energy between 12 meV and 17 meV that is in good agreement with our activation energies. They attributed this donor to Si atoms substituting for Ga in the GaN lattice (Si_Ga_) and justified the range of activation energies to different levels of donor concentrations and acceptor compensation in the samples. Their PL measurements yielded the position of the optical Si donor level in the band gap at 22(4) meV below the conduction band. Another deeper donor level with an activation energy around 34 meV was also present and was assigned to oxygen donors substituting for nitrogen (O_N_). In earlier publications, the unintentional n-type conductivity that has often been observed in as-grown GaN has commonly been attributed to nitrogen vacancies (V_N_) but first-principle calculations performed by Van De Walle *et al*.^19^ have shown that the high formation energy of nitrogen vacancies under n-type conditions makes it very unlikely that they would form spontaneously during growth of not-intentionally doped GaN, and hence they cannot be responsible for n-type conductivity. Their calculations together with experimental evidence that unintentionally doped n-type GaN samples contained concentrations of extrinsic donors high enough to explain the observed electron concentrations are enough to confirm that the unintentional n-type conductivity is due to unintentional incorporation of donor impurities (particularly oxygen), reinforcing the conclusions made by Götz and colleagues. Nonetheless, it must be noted that purposely created nitrogen vacancies (e.g., during irradiation or ion implantation) will increase the electron concentration as well. In the present work, after implantation and subsequent annealing, remaining nitrogen vacancies cannot be excluded even if diluted enough being not observable nearby the Ta probe atoms. Furthermore, contrary to n-type conditions, nitrogen vacancies have a low formation energy in p-type GaN, making them a likely compensating center in the case of acceptor doping^19^.

Regarding Zn-doped GaN, it has already been shown in several works that Zn substitutes for Ga (Zn_Ga_) and introduces an acceptor level around 350 meV above the top of the valence band maximum^10–12^. It is established that Zn_Ga_ is responsible for the blue luminescence peaking at 2.9 eV that is often observed in PL spectroscopy, not only on Zn-doped GaN samples but also in undoped and Si-doped^11,12^. This blue band is attributed to transitions from the conduction band (or shallow donors at very low temperatures) to the Zn_Ga_ acceptor. Zn-doped GaN also shows the presence of unintentionally introduced O_N_ shallow donors with ionization energies in the range of 20 meV to 30 meV^20^ (similar to the n-type doped samples) but by adding a sufficient concentration of Zn, it is possible to closely compensate the donors and thus render the material insulating^10^.

**Figure 9:**
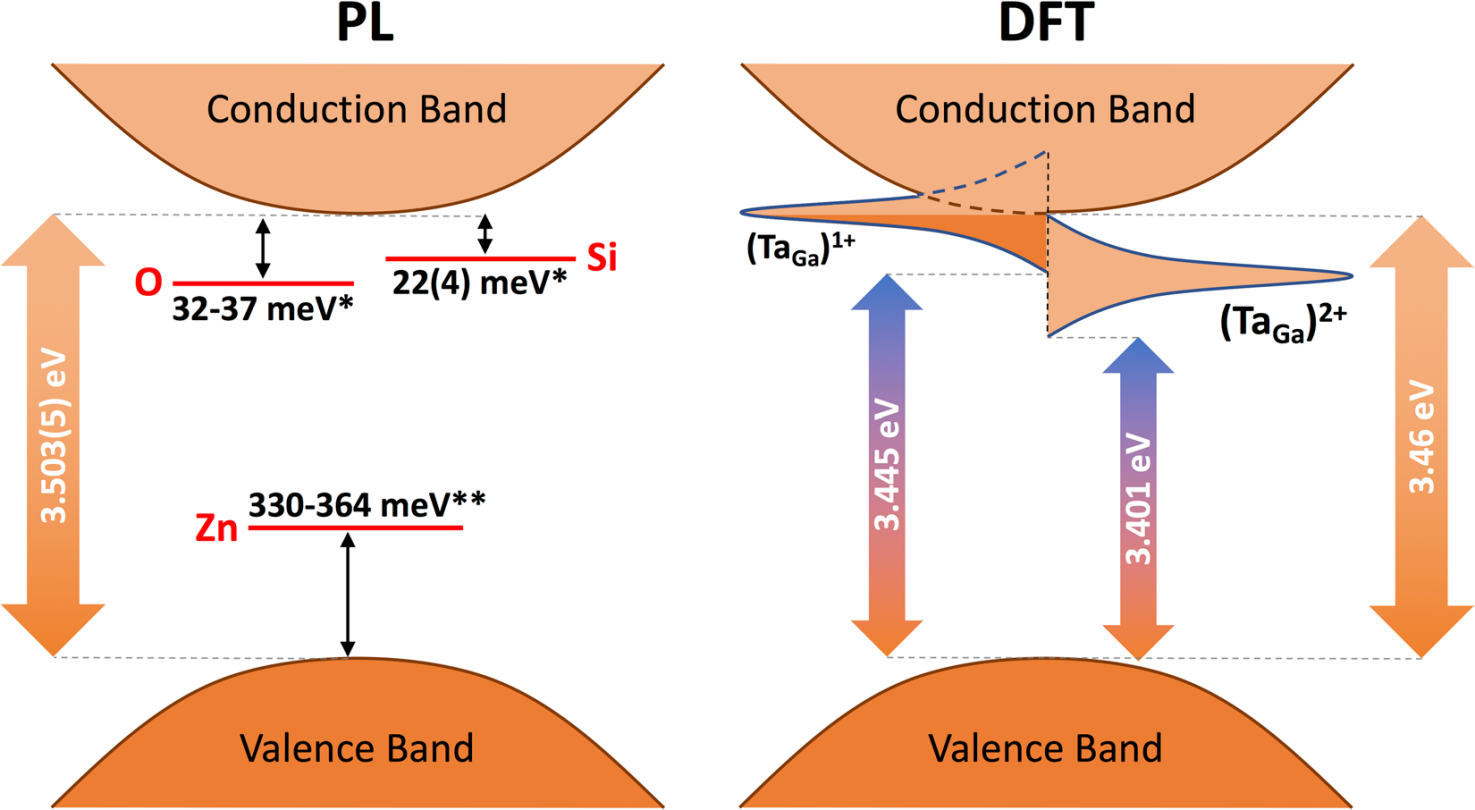
GaN band diagram including inside the band gap the impurity bands from the simulated DOS of ${{\rm{Ta}}}_{{\rm{Ga}}}^{1+}$ and ${{\rm{Ta}}}_{{\rm{Ga}}}^{2+}$ and the reported bands associated to Si, Zn, and O dopants/defects. For the ${{\rm{Ta}}}_{{\rm{Ga}}}^{1+}$ state, the band is partially filled. *From ref.^9^, **from refs^10–12^.

In Fig. 9 it is possible to see a band diagram including the impurity bands from the simulated DOS of ${{\rm{Ta}}}_{{\rm{Ga}}}^{1+}$ and ${{\rm{Ta}}}_{{\rm{Ga}}}^{2+}$ and the bands from the previously mentioned defects in GaN.

**Figure 10:**
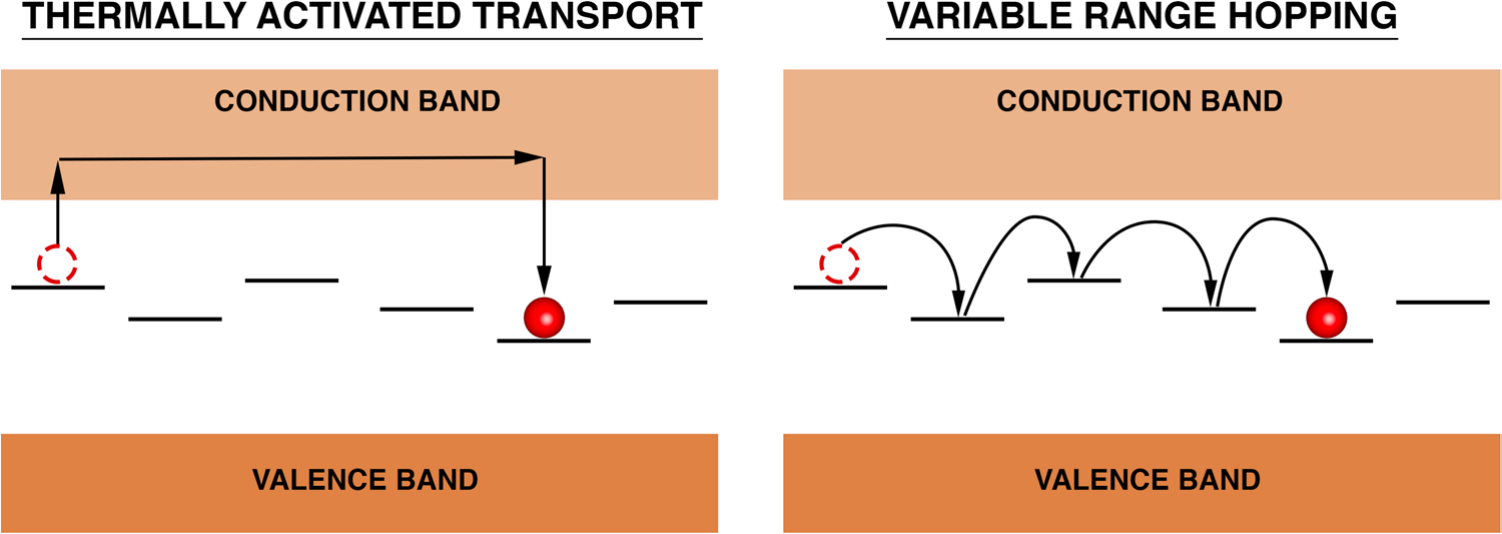
Schematics illustrating the two models used in this study to explain how the probe atoms recover electrons from the GaN host: thermally activated transport or variable-range hopping. In the first, electrons are thermally activated to the conduction band and then trapped at the PAC probe. This mechanism is dominant at high temperature. Variable range hopping allows the tunneling of electrons between defect states even at low temperature.

We can now have a glimpse of what is being observed in the *e*^−^-*γ* PAC measurements, focusing primarily on the GaN:Si sample. The electron that is lost by the Ta probe during the internal conversion decay provokes a cascade of ejected Auger electrons that leaves the atom in a highly electron deficient/unstable state (it can be estimated that after the emission of a L-shell electron, Ta loses on average around 9 electrons in the entire process^21^). This disturbance takes place in the picoseconds scale, therefore when the PAC measurement starts, the holes left by the Auger electrons are usually already located in the valence band. However, due to the probabilistically different Auger emission and ionization of different Ta probes, as well as the random availability of electrons in their vicinity, the recombination is unpredictable. Initially some of the probes are not fully recovered electronically and thus the initial state (State 1) is described by a broad EFG distribution. After being fully recovered, all probes are in the final stable state, ${{\rm{Ta}}}_{{\rm{Ga}}}^{2+}$, with the Ta impurity band completely unfilled. The electrons needed for the electronic recovery come from the Si_Ga_ donors (and most probably from O_N_ and V_N_ as well), hence the obtained activation energy for this thermally activated process at higher temperatures is in accordance with the activation energy previously associated to Si_Ga_ shallow donors from Hall effect measurements. For the measurements between 120 K and 294 K, the intermediate state corresponding to ${{\rm{Ta}}}_{{\rm{Ga}}}^{1+}$ is also visible before the full transition to ${{\rm{Ta}}}_{{\rm{Ga}}}^{2+}$. We suspect that this is due to the impurity band of ${{\rm{Ta}}}_{{\rm{Ga}}}^{2+}$ being slightly lower in energy than the Si_Ga_ impurity band (see Figs 7 and 9), therefore some of the donor electrons that are not yet thermally excited to the conduction band will tunnel to the Ta probes and hence both Ta charge states are visible. The extra electron in ${{\rm{Ta}}}_{{\rm{Ga}}}^{1+}$ will ultimately go to the conduction band and so only the ${{\rm{Ta}}}_{{\rm{Ga}}}^{2+}$ is seen as the final state. The higher the temperature, the faster the electronic recovery is and the faster all donor electrons are excited to the conduction band, therefore at higher temperatures only the ${{\rm{Ta}}}_{{\rm{Ga}}}^{2+}$ state is visible. In the low temperature regime, not only the transition rates are much lower but also only the ${{\rm{Ta}}}_{{\rm{Ga}}}^{2+}$ state is visible. This is due to the fact of holes in the valence band being easily recovered by donor electrons even at low temperatures (hence the ${{\rm{Ta}}}_{{\rm{Ga}}}^{2+}$ state is easily achieved) but the energy is probably not enough for an extra electron to tunnel from a Si_Ga_ donor to a Ta probe to produce the ${{\rm{Ta}}}_{{\rm{Ga}}}^{1+}$ state. Also, since the transition rates at lower temperatures deviate from the high temperatures regime, the main mechanism for charge carrier transport in this regime might possibly no longer be thermally activated but instead be better described by a Variable Range Hopping (VRH) mechanism (Fig. 10). This hypothesis is tested on the inset of Fig. 8 where the characteristic ${T}^{-1/4}$ dependence of VRH for the transition rate is fitted. With the reported experimental errors and few experimental data points, the exact model cannot be fully confirmed, but since the transition rates in this regime are much above the expected values if obtained from the simple thermal activation process observed at higher temperatures, we expect that defects can then predominantly contribute to the charge carrier transport and, consequently, to the transition rates, in a typical VRH process.

Going from the GaN:Si sample to the GaN:Zn sample, the presence of unintended donor defects (including Si-donors) besides the intentionally introduced Zn dopants is expected, as previously mentioned, and since the results for both samples are very similar, the majority of the physical processes involved are expected to be the same. The activation energy obtained in the higher temperatures regime is very close to the GaN:Si sample but the biggest difference is the downshift to lower amplitudes in the Arrhenius plot. This can be understood by the fact that the Zn_Ga_ deep acceptor band compensates some of the electrons provided by the shallow donors. Therefore, in spite of the available electrons for the electronic recovery having the same origin as in the GaN:Si sample (therefore similar activation energy), the number of available electrons is much lower, hence the downshift in the Arrhenius plot. In fact, for GaN:Si at 294 K the ${{\rm{Ta}}}_{{\rm{Ga}}}^{1+}$ state is inclusively more visible than the ${{\rm{Ta}}}_{{\rm{Ga}}}^{2+}$ state due to the high number of non-ionized Si_Ga_ donors (thus more electrons available to tunnel to the Ta probes), but that same state is not so visible in GaN:Zn at the same temperature due to the high number of donors compensated by the Zn_Ga_ acceptors and lower donor concentration.

One final note must be made regarding the fact of the transition rate from the intermediate state (${{\rm{Ta}}}_{{\rm{Ga}}}^{1+}$) to the final state (${{\rm{Ta}}}_{{\rm{Ga}}}^{2+}$) (transition rate $2\to 3$ in Table 2) to be essentially constant as a function of temperature. Since the impurity band of ${{\rm{Ta}}}_{{\rm{Ga}}}^{1+}$ is in the conduction band minimum (Figs 7 and 9), the activation energy corresponding to this transition is very low when compared to the other transitions (in fact it should be very close (or equal) to 0 eV). This means that the variation of this transition rate with temperature is much smaller than the variation of the other transition rates, so the fitting can be done considering this transition to be constant in temperature within the experimental error. The inverse of the transition rate can be used to estimate the *mean life* time of the ${{\rm{Ta}}}_{{\rm{Ga}}}^{1+}$ state^15^, which is equal to $1/(38(3)\,{\rm{MHz}})\approx 26(2)\,{\rm{ns}}$ for GaN:Si and $1/(42(5)\,{\rm{MHz}})\approx 24(3)\,{\rm{ns}}$ for GaN:Zn.

This entire process observed in ${e}^{-}$-$\gamma$ PAC does not occur in $\gamma$-$\gamma$ PAC because there is no loss of inner orbit electrons during the decay in the latter case. After the decay of ^181^Hf to ^181^Ta there is a first state of 17.8 μs (see Fig. 1) long enough for the probes to fully recombine the electronic states and relax to their stable position with the electronic state ${{\rm{Ta}}}_{{\rm{Ga}}}^{2+}$ before the PAC cascade occurs. During the $\gamma$-$\gamma$ PAC measurement only the most stable state ${{\rm{Ta}}}_{{\rm{Ga}}}^{2+}$ is observed (having no relevant variations with temperature), hence a single EFG is present. We suspect that the ${{\rm{Ta}}}_{{\rm{Ga}}}^{1+}$ state visible at some temperatures in ${e}^{-}$-$\gamma$ PAC is favored in that situation due to the avalanche of electrons that are lost in the internal conversion decay, thus turning the probes into very strong Coulomb attractors that increase the probability of one electron to go from a donor to a Ta probe even after all the needed electrons are recovered to achieve the ${{\rm{Ta}}}_{{\rm{Ga}}}^{2+}$ state, which is not the case in $\gamma$-$\gamma$ PAC. This premise is reinforced by the higher expression of the ${{\rm{Ta}}}_{{\rm{Ga}}}^{1+}$ state in the Si-doped sample when compared to the Zn-doped sample, where the former has more electron donors available than the latter.

## Discussion

The combination of the ${e}^{-}$-$\gamma$ and $\gamma$-$\gamma$ PAC techniques after implantation and annealing of ^181^Hf/^181^Ta probes into Si- and Zn-doped GaN samples allowed performing a detailed study of the Ta impurity dopant at a Ga site and the characterization of different localized electronic states, as a function of temperature and sample doping.

The $\gamma$-$\gamma$ PAC measurements showed a single stable state that was attributed to the probe state ${{\rm{Ta}}}_{{\rm{Ga}}}^{2+}$ with no relevant changes with temperature. On the other hand, the ${e}^{-}$-*γ* PAC measurements showed not only the presence of the stable state ${{\rm{Ta}}}_{{\rm{Ga}}}^{2+}$ but also the presence of the metastable state ${{\rm{Ta}}}_{{\rm{Ga}}}^{1+}$ at some temperatures, including strong temperature-dependent variations. Such detailed observations are only possible due to the creation of a localized highly ionized Ta state after the loss of electrons triggered by the internal conversion decay and the underlying processes for their recovery.

A comparison of the density of states (DOS) for the simple cell of GaN to the supercells with the different charged states of Ta (${{\rm{Ta}}}_{{\rm{Ga}}}^{0}$, ${{\rm{Ta}}}_{{\rm{Ga}}}^{1+}$ and ${{\rm{Ta}}}_{{\rm{Ga}}}^{2+}$) was also done. It was possible to see that Ta induces an impurity band adjacent to the conduction band that is semi-filled for ${{\rm{Ta}}}_{{\rm{Ga}}}^{0}$ and ${{\rm{Ta}}}_{{\rm{Ga}}}^{1+}$ but it is completely empty for ${{\rm{Ta}}}_{{\rm{Ga}}}^{2+}$. In fact, integrating the filled part of the impurity band gives 1.9 electrons for ${{\rm{Ta}}}_{{\rm{Ga}}}^{0}$ and 1.0 electrons for ${{\rm{Ta}}}_{{\rm{Ga}}}^{1+}$. This is in accordance with the oxidation state of Ga at that site (3+) and the preferred oxidation state of Ta (5+), therefore being expected that Ta at a Ga site shares three valence electrons for the chemical bonding with the neighboring N atoms and gives away two valence electrons to the conduction band, which was indeed observed in this work.

Regarding the temperature dependence of the transition rates in the ${e}^{-}$-$\gamma$ PAC measurements, two different regimes were observed. At higher temperatures, a thermally activated process was observed for the electronic recombination with activation energies of 15(2) meV and 12(1) meV for the GaN:Si and GaN:Zn samples, respectively, which were associated to Si_Ga_ shallow donors in both samples (and probably having some contributions from unintended O_N_ and V_N_ defects). However, the Arrhenius plot of GaN:Zn is downshifted in comparison to GaN:Si due to donors compensated by the Zn acceptors, hence reducing the number of available electrons during the electronic recovery. At lower temperatures, it is suggested that the recombination process occurs via Variable Range Hopping instead of via a thermally activated process. Nonetheless, the experimental errors do not allow for a completely verified model solution in this regime. The two relevant electron processes are schematically described in Fig. 10. At high temperatures the dominant charge carrier transport is thermally activated by exciting donor electrons into the conduction band. At low temperatures the variable range hopping seems more appropriate where electrons tunnel via multiple defect states.

Given the amount and the quality of the information that was possible to obtain about the studied samples and, more importantly, how it was possible to distinguish the doping characteristics from one sample to another, we believe that this work demonstrates the potential use of ${e}^{-}$-$\gamma$ PAC combined with $\gamma$-$\gamma$ PAC for the study of electronic properties of materials and/or samples where, for instance, Hall effect measurements are difficult/impossible to perform due to small dimensions or low-quality ohmic contacts.

## Methods

### Perturbed angular correlations theory

In a Perturbed Angular Correlation experiment, a radioactive probe which decays in a double cascade (emitting two photons, ${\gamma }_{1}$ and ${\gamma }_{2}$) is introduced in a sample by implantation, diffusion or neutron activation. The hyperfine interaction of the electric field gradient (EFG) at the probe’s site with the electric quadrupole moment of the intermediate level of the cascade causes a time-dependent perturbation in the angular dependence of the emission probability of ${\gamma }_{2}$ with respect to ${\gamma }_{1}$. Since the EFG is a traceless matrix and diagonal in its principal axis, it can be completely described by only two parameters: the ${V}_{zz}$ component and the axial asymmetry parameter $\eta =({V}_{xx}-{V}_{yy})/{V}_{zz}$, considering that $|{V}_{xx}|\le |{V}_{yy}|\le |{V}_{zz}|$^22^. The time-dependent oscillations in the anisotropic emission of ${\gamma }_{2}$ then define the observable frequency ${\omega }_{0}$:

1 $${\omega }_{0}=k\frac{eQ{V}_{zz}}{4I(2I-1)\hslash }$$

where $I$ and $Q$ are the spin and the electric quadrupole moment of the intermediate level of the cascade, respectively, and $k=3$ (or 6) for integer (or half-integer) spin^22^.

The coincidence spectra $N(\theta ,t)$ can be recorded, where $\theta$ is the angle between detectors and $t$ is the time delay between the detection of ${\gamma }_{1}$ and ${\gamma }_{2}$, allowing for the experimental perturbation function $R(t)$ to be calculated:

2 $$R(t)=2\frac{N(180^\circ ,t)-N(90^\circ ,t)}{N(180^\circ ,t)+2N(90^\circ ,t)}$$

Each $N(\theta ,t)$ is represented by a sum proportional to the perturbation factor ${G}_{{k}_{1}{k}_{2}}^{{N}_{1}{N}_{2}}(t)=\sum _{n}\,{S}_{{k}_{1}{k}_{2},n}^{{N}_{1}{N}_{2}}\,\cos ({\omega }_{n}t)$, so it is described by a sum of oscillatory terms with frequencies ${\omega }_{n}$. For a single EFG, a spin $I=5/2$ yields a triplet of frequencies ${\omega }_{1}$, ${\omega }_{2}$ and ${\omega }_{3}={\omega }_{1}+{\omega }_{2}$ with ${\omega }_{n}={C}_{n}(\eta ){\omega }_{0}$, so three peaks will be observed in the Fourier transform of the perturbation function^23^.

In the case of fluctuating EFGs, the theory based on stochastic processes applied to PAC developed by Winkler and Gerdau^15^ is considered. In their formalism, the time-evolution operator is given by $\hat{\Omega }(t)=\exp [(\,-\frac{i}{\hslash }{H}_{st}^{\times }+\hat{R})t]$, where $(\,-\frac{i}{\hslash }{H}_{st}^{\times }+\hat{R})$ is called the Blume matrix and ${H}_{st}^{\times }$ and $\hat{R}$ are Liouville operators, the former being constructed from the Hamiltonians that describe the different possible states (each one described by a static EFG) and the latter containing the transition rates between the different possible states. In this case, the observable perturbation factor is given by

3 $${G}_{{k}_{1}{k}_{2}}^{{N}_{1}{N}_{2}}(t)=\sum _{n}\,{S}_{{k}_{1}{k}_{2},n}^{{N}_{1}{N}_{2}}\,\cos ({\omega }_{n}t)\,\exp (\,-\,{\lambda }_{n}t)$$

where the amplitudes ${S}_{{k}_{1}{k}_{2},n}^{{N}_{1}{N}_{2}}$ depend on the initial percentage at $t=0$ of each individual state and on the eigenvectors of the Blume matrix, being $-{\lambda }_{n}+i{\omega }_{n}$ the eigenvalues. The real components of the eigenvalues are always negative and are only non-zero for non-zero transition rates, thus the damping induced by them in the oscillations of the perturbation function are a signature of the presence of dynamic processes.

To account for the fact that probes in equivalent sites might have slight deviations in their EFGs (e.g., due to possible remaining diluted defects not preferentially attached to them), a Lorentzian distribution characterized by their central frequency ${\omega }_{0}$ and full width at half maximum (FWHM) is integrated for each EFG individually in the perturbation function.

Finally, in the case of ${e}^{-}$-$\gamma$ PAC, the anisotropy terms ${A}_{k}(\gamma )$ being multiplied to the perturbation factor ${G}_{{k}_{1}{k}_{2}}^{{N}_{1}{N}_{2}}(t)$ in the construction of the coincidence spectra $N(\theta ,t)$ must be changed to ${A}_{k}({e}^{-})={b}_{k}({e}^{-}){A}_{k}(\gamma )$ whilst the underlying theory remains the same^24,25^. The values for particle parameter ${b}_{k}({e}^{-})$ can be found in tables^25^.

### Perturbed angular correlation experiments

Two commercially available (from Technologies and Devices International Inc., 12214 Plum Orchard Drive, Silver Spring, MD 20904, USA) ~11 μm thick GaN samples were grown by hydride vapor phase epitaxy on sapphire substrates and doped with Si (concentration ${N}_{d}-{N}_{a}=(1\,-\,3)\times {10}^{18}\,{{\rm{cm}}}^{-3}$) and Zn (high resistivity $> 1\times {10}^{5}\,\Omega \,{\rm{cm}}$), respectively. ^181^Hf/^181^Ta probes were implanted at room temperature in each sample along the surface normal at the isotope separator of the University of Bonn with 160 keV energy to typical fluences of $\sim \,{10}^{13}\,{\rm{atoms}}/{{\rm{cm}}}^{2}$. Then, the samples were annealed in a rapid thermal annealing apparatus between graphite strips under nitrogen flow with a holding time of 120 s at 950 °C and using a GaN proximity cap, as previously described to be optimal for GaN^14^. The $\gamma$-$\gamma$ PAC experiments were carried out on a 6-LaBr_3_ detector spectrometer^26^ while the ${e}^{-}$-$\gamma$ PAC experiments were carried out on a P. Kleinheinz and K. Siegbahn spectrometer equipped with two magnetic lenses and two BaF_2_ detectors^2,3^. Both $\gamma$-$\gamma$ and ${e}^{-}$-$\gamma$ PAC measurements were performed as a function of temperature, between room temperature and 10 K using closed cycle He refrigerators. ${e}^{-}$-$\gamma$ PAC experiments were further performed (under vacuum) from room temperature up to ~500 K using a sample holder heating system. During the ${e}^{-}$-$\gamma$ measurements, the samples were oriented such that the normal surface axis (corresponding to the c-axis of the wurtzite lattice) was in the detectors’ plane with 45° in between detectors’ axis. The $\gamma$-$\gamma$ measurements were performed in a 6-detector PAC spectrometer where the sample cannot be mounted establishing a high symmetry orientation with all detector pairs. This leads to a mixture of different and undetermined relative orientations for different pairs of detectors. Still, that only affects the relative amplitudes of the triplet of frequencies characterizing the same EFG at the observable R(t) function and do not affect the associated frequencies from where the hyperfine fields are determined.

### Density functional theory simulations

The simulations were performed via the full-potential (linearized) augmented plane wave plus local orbitals method [FP-(L)APW+lo] as implemented in the WIEN2k code^27^. The Perdew, Burke and Ernzerhof parameterization (GGA-PBE)^27^ was considered as exchange-correlation functional for the structural optimization and calculation of the electric field gradients, and the modified Becke-Johnson exchange potential (mBJ)^28^ was used for the calculation of the density of states, band structure and band gap. The wurtzite structural parameters of GaN as found in the work of Miwa *et al*.^6^ were considered ($a=3.146\,{\rm{\AA }}$, $b=3.146\,{\rm{\AA }}$, $c=5.125\,{\rm{\AA }}$) with the internal atomic positions being optimized by minimizing the atomic forces to a maximum limit of 2 mRy/bohr in a self-consistent way, where a value of $u=0.377$ was obtained for the internal parameter, which corresponds to the value reported experimentally by Schulz and Thiermann^29^ and from previous simulations^17^. To simulate an isolated Ta impurity, a 3 × 3 × 2 supercell of GaN was constructed. Its size was determined by increasing the supercell until the variation of the EFG at the Ta site was in the same order of magnitude of the experimental error in the PAC measurements. A cut-off value for the plane wave expansion of Rmt * Kmax = 7.0 was considered, where Rmt is the muffin-tin sphere radius and Kmax is the largest K-vector of the plane wave expansion of the wave function. 76 and 24 k-points in the irreducible Brillouin zone were used for the GaN simple cell and for the 3 × 3 × 2 supercell with the Ta impurity, respectively. Different charge states were considered for the supercell with Ta probes, where removed charges were compensated by adding a homogeneous background of opposite charge to keep the entire cell in a neutral state^27,30,31^. For instance, if an electron is removed from the cell, the extra positive charge (the hole) can be localized but a uniform negative charge will maintain the neutrality of the cell whilst not resulting in any extra interactions.
